# Sirtuin-3 Is Expressed by Enteric Neurons but It Does not Play a Major Role in Their Regulation of Oxidative Stress

**DOI:** 10.3389/fncel.2016.00073

**Published:** 2016-03-22

**Authors:** Rebecca K. Bubenheimer, Isola A. M. Brown, David E. Fried, Jonathon L. McClain, Brian D. Gulbransen

**Affiliations:** ^1^Neuroscience Program, Michigan State UniversityEast Lansing, MI, USA; ^2^Department of Physiology, Michigan State UniversityEast Lansing, MI, USA; ^3^Pharmacology and Toxicology Program, Michigan State UniversityEast Lansing, MI, USA

**Keywords:** Sirt3, sirtuin, autonomic, intestine, peripheral nervous system, oxidative stress

## Abstract

Gut inflammation contributes to the development of gut motility disorders in part by disrupting the function and survival of enteric neurons through mechanisms that involve oxidative stress. How enteric neurons regulate oxidative stress is still poorly understood. Importantly, how neuron autonomous antioxidant mechanisms contribute to the susceptibility of enteric neurons to oxidative stress in disease is not known. Here, we discover that sirtuin-3 (Sirt3), a key regulator of oxidative stress and mitochondrial metabolism, is expressed by neurons in the enteric nervous system (ENS) of the mouse colon. Given the important role of Sirt3 in the regulation of neuronal oxidative stress in the central nervous system (CNS), we hypothesized that Sirt3 plays an important role in the cell autonomous regulation of oxidative stress by enteric neurons and that a loss of Sirt3 increases neuronal vulnerability during intestinal inflammation. We tested our hypothesis using a combination of traditional immunohistochemistry, oxidative stress measurements and *in vivo* and *ex vivo* measures of GI motility in healthy and inflamed wild-type (*wt*) and Sirt3 null (*Sirt3*^−/−^) mice. Our results show that Sirt3 is widely expressed by neurons throughout the myenteric plexus of the mouse colon. However, the deletion of Sirt3 had surprisingly little effect on gut function and susceptibility to inflammation. Likewise, neither the genetic ablation of *Sirt3* nor the inhibition of Sirt3 with antagonists had a significant effect on neuronal oxidative stress. Therefore, we conclude that Sirt3 contributes very little to the overall regulation of neuronal oxidative stress in the ENS. The functional relevance of Sirt3 in enteric neurons is still unclear but our data show that it is an unlikely candidate to explain neuronal vulnerability to oxidative stress during inflammation.

## Introduction

The enteric nervous system (ENS) controls essential gastrointestinal (GI) reflexes such as patterns of movement (i.e., peristalsis), fluid exchange across the intestinal mucosa and local blood flow (Furness, 2012). This becomes most evident in disease when gut functions are impaired by changes to the structure or neurochemistry of the ENS. Indeed, alterations to the survival or function of enteric neurons are associated with impaired gut function in a number of conditions such as old age (Wade and Cowen, 2004; Camilleri et al., 2008), diabetes (Chandrasekharan et al., 2011), inflammatory bowel diseases (IBD; Gulbransen et al., 2012), achalasia (De Giorgio et al., 1999), chronic intestinal pseudo-obstruction (Antonucci et al., 2008), slow-transit constipation (De Giorgio et al., 2004), Parkinson’s disease (PD; Wakabayashi et al., 1988; Beach et al., 2010; Devos et al., 2013), irritable bowel syndrome (IBS; Lindberg et al., 2009; Ohlsson et al., 2010) and infectious diseases (Coron et al., 2009; Lourenssen et al., 2010).

Oxidative stress is one of the most important mechanisms that contributes to ENS dysfunction in disease (Lih-Brody et al., 1996; Thrasivoulou et al., 2006; Chandrasekharan et al., 2011; Roberts et al., 2013; Brown et al., 2016). For example, the inability to buffer reactive oxygen species is considered an important factor in the susceptibility of inhibitory, nitrergic neurons to injury in conditions such as diabetes (Chandrasekharan et al., 2011) and inflammation (Sido et al., 1998; Esworthy et al., 2001; Rezaie et al., 2007; Zhu and Li, 2012; Roberts et al., 2013) and excitatory, cholinergic neurons in advanced age (Wade, 2002; Camilleri et al., 2008; Korsak et al., 2012; Saffrey, 2013). Neurons continually generate reactive oxygen species during normal functions because of their high energy demand to sustain electrical signaling. Yet neurons express few anti-oxidant defenses and have a low ability to cope with reactive oxygen species. Therefore, even a subtle shift in the limited defense mechanisms that neurons do possess could have major effects on their susceptibility to disease. Despite the importance of oxidative stress in neuronal susceptibility to cell injury in many enteric neurodegenerative conditions, little is known about the factors that regulate oxidative stress in enteric neurons.

Sirtuin-3 (Sirt3) is a member of the sirtuin family of nicotinamide adenine nucleotide (NAD)–dependent deacetylases that has emerged as an important regulator of neuronal survival in times of stress (Schwer et al., 2002; Kim et al., 2011; Weir et al., 2012; Chen et al., 2013; Dai et al., 2014). In cultured neurons, the expression of Sirt3 is both necessary (Kim et al., 2011) and sufficient (Kim et al., 2011; Shulyakova et al., 2014) for neuroprotection in models of oxidant-induced neurotoxicity and excitotoxicity. These neuroprotective actions appear to stem mainly from Sirt3’s ability to activate key scavengers of reactive oxygen species in mitochondria (Shi et al., 2005; Sundaresan et al., 2009; Kim et al., 2010). Indeed, mice deficient in Sirt3 exhibit higher levels of oxidative stress (Chen et al., 2013) and display enhanced susceptibility to metabolic diseases (Hirschey et al., 2011).

Given the important role of Sirt3 in the regulation of neuronal oxidative stress in neurons from the central nervous system (CNS), we hypothesized that Sirt3 is an important regulator of oxidative stress in the ENS. We tested our hypothesis using a combination of traditional immunohistochemistry, oxidative stress measurements and *in vivo* and *ex vivo* measures of GI motility in wild-type (*wt*) and Sirt3 null (*Sirt3*^−/−^) mice. Our results show that Sirt3 is widely expressed by myenteric neurons in the mouse colon. However, the deletion of Sirt3 has surprisingly little effect on gut function and the susceptibility to inflammation. Likewise, neither the genetic ablation of Sirt3 nor its inhibition with antagonists has a significant effect on neuronal oxidative stress. Our results provide the first evidence of Sirt3 expression in the ENS. However, the main function of Sirt3 in enteric neurons is unclear because it does not appear to play a major role in the regulation of oxidative stress by enteric neurons.

## Materials and Methods

### Animals

Experimental protocols were reviewed and approved by the Institutional Animal Care and Use Committee at Michigan State University. Sirt3 null mice (129-*SIRT3^tm1.1Fwa^*/J; RRID:IMSR_JAX:012755), hereafter referred to as *Sirt3*^−/−^(Lombard et al., 2007), and background controls (129S1/SvImJ; RRID:IMSR_JAX:002448), hereafter referred to as wild-type (*wt*; The Jackson Laboratory, Bay Harbor, ME, USA), of both sexes (8–10 weeks of age) were used for all experiments unless otherwise stated. Male C57BL/6 mice (8–10 weeks of age) from Charles River Laboratories (Hollister, CA, USA) were used in some experiments to test Sirt3 expression in inflammation and to compare oxidative stress levels of myenteric neurons. Mice were maintained in a temperature controlled 12 h light:12 h dark cycle environment with *ad libitum* access to food and water. All genotyping was performed by the Research Technology Support Facility at Michigan State University using the standard PCR protocol supplied by Jackson Labs for *Sirt3*^−/−^ mice (information available at www.jax.org).

### *In Vivo* Measures of Colonic Motility

#### Pellet Production

Mice were individually housed in clean, empty cages and fecal output was monitored for 1 h beginning at Zeitgeber time + 3 for 5 consecutive days. Mice were acclimatized over the first 2 days and data was recorded over the last 3 days. Pellets were collected as soon as they were produced and counted and weighed to obtain a wet weight. Pellets were then dried overnight to determine dry weight.

#### Colon Bead

Individually housed animals were fasted overnight and lightly anesthetized with isoflurane. A 2 mm bead was inserted 2 cm into the colon and the time to expulsion recorded.

### *In Vivo* Colitis

We used the dinitrobenzene sulfonic acid (DNBS) model to induce colitis in mice (Gulbransen et al., 2012). Anesthetized mice received an enema of DNBS (5.5 mg/mouse in 0.1 mL 50% ethanol/50% saline) via a gavage needle inserted 3 cm into the colon. Control animals received an enema of saline. We monitored animal weight daily and 48 h after treatment, animals were euthanized via cervical dislocation to collect tissue samples. The extent of inflammation was scored using an established macroscopic damage scoring system (Storr et al., 2009).

### Contractility Studies

Longitudinally oriented segments of mouse intestine were mounted between two platinum ring stimulating electrodes in organ baths. Each intestinal segment was secured to a tissue holder and connected to a force-displacement transducer (Grass Instruments, Quincy, MA, USA). Frequency-response curves (2–30 Hz) for neurogenic contractions and relaxations were generated by electrical field stimulation (EFS) supplied through the electrodes by a GRASS stimulator (S88, GRASS telefactor, West Warwick, RI, USA) and data was charted with Labscribe (iWorx, Dover, NH, USA). Tissue segments were placed under 0.5 g initial tension and allowed to equilibrate for 20 min. Neurogenic relaxations were studied in tissues precontracted with 5 μM prostaglandin F2-α (PGF_2α_). Bethanechol (BCH, 10 μM) was used to assess the maximum contractile response and tetrodotoxin (TTX, 0.3 μM) was applied to block neurogenic responses.

### Whole-Mount Immunohistochemistry

Whole-mount preparations of colonic myenteric plexus and longitudinal muscle (LMMP) were prepared from tissue fixed in Zamboni’s fixative (Nasser et al., 2007) or 4% paraformaldehyde (PFA; Joseph et al., 2011). Nonspecific labeling was blocked by incubating tissue for 45 min in a solution containing 4% normal donkey serum or goat serum, 0.4% Triton X-100, and 1% bovine serum albumin. Primary antibodies were applied overnight at room temperature and secondary antibodies were applied for 2 h at room temperature. Antibody details are supplied in Table 1. Images were acquired through the 40X objective (Planfluor, 0.75 numerical aperture) of an upright epifluorescence microscope (Nikon Eclipse Ni, Melville, NY, USA) with a Retiga 2000R camera (QImaging, Surrey, BC, Canada) controlled by QCapture Pro 7.0 software (QImaging) or by confocal imaging using the Plan-Aprochromat 60X oil immersion objective (1.42 numerical aperture) of an inverted Olympus Fluoview FV1000 microscope (Olympus, Center Valley, PA, USA). Software settings were established using the immunoreactivity of each antibody in control tissues and then kept constant for all remaining tissues.

**Table 1:** **Antibodies used in this study**.

Antibody	Source	Dilution
**Primary antibodies**
Goat anti-Calretinin	Swant, Switzerland; CG1	1:1000
Guinea Pig anti-CGRP	Peninsula Labs, San Carlos, CA, USA; T-5053	1:500
Chicken anti-GFAP	Abcam, Cambridge, MA, USA; ab4674	1:1000
HuC/D Biotinylated	Invitrogen, Carlsbad, CA, USA; A-21272	1:200
Sheep anti-nNos	Millipore, Darmstadt, Germany; ab1529	1:500
Rabbit anti-Sirt3	Millipore, Darmstadt, Germany; 07-1596	1:100
Rabbit anti-Sirt3	Cell Signaling, Danvers, MA, USA; 5490	1:200
Rabbit anti-Sirt3	Abcam, Cambridge, MA, USA; Ab86671	1:200
**Secondary antibodies**		
Donkey anti-Rabbit 488	Jackson, Bay Harbor, ME, USA; 711-545-152	1:400
Goat anti-Rabbit 488	Lifetech, Carlsbad, CA, USA; A-11034	1:400
Streptavidin 488	Jackson, Bay Harbor, ME, USA; 016-540-084	1:400
Donkey anti-Goat 568	Invitrogen, Carlsbad, CA, USA; A-11057	1:400
Donkey anti-Sheep 568	Invitrogen, Carlsbad, CA, USA; A-21099	1:400
Goat anti-Chicken 568	Invitrogen, Carlsbad, CA, USA; A-11041	1:400
Donkey anti-Guinea Pig 594	Jackson, Bay Harbor, ME, USA; 706-585-148	1:400
Streptavidin 594	Jackson, Bay Harbor, ME, USA; 016-580-084	1:400

### Thiol Oxidation

We quantified neuronal thiol oxidation as a measure of oxidative stress as previously described (Mullett et al., 2013). Briefly, reduced (-SH) and oxidized (-SS) thiols were labeled with 1 μM Alexa Fluor 680 C_2_ maleimide and 1 μM Alexa Fluor 546 C_5_ maleimide, respectively. Dyes were dissolved in 4% PFA, 0.02% Triton X-100 and 1 mM N-ethylymaleimide in PBS. Tissue was washed in 5 mM tris(2-carboxyethyl)phosphine hydrochloride to convert oxidized thiols to reduced thiols. Images were obtained by epifluorescence microscopy as described above and the ratio of 546-maleimide/680-maleimide (SS/SH) calculated with ImageJ software (NIH).

### Drugs/Chemicals

Tenovin-6 was purchased from Cayman Chemicals (Ann Arbor, MI, USA). All other reagents were purchased from Sigma-Aldrich (St. Louis, MO, USA).

### Data Analysis

Cell counts were performed using the cell counter plug-in of ImageJ software. Individual neuron subpopulations are presented as a percentage of total enteric neuron numbers defined by HuC/D-immunoreactivity. Enteric neuron numbers are presented as the ganglionic neuronal packing density. We calculated the neuron packing density per ganglion by using GFAP-immunoreactivity to demarcate the borders of each ganglion and HuC/D-immunoreactivity to count the number of neurons within that two-dimensional area. Ganglionic GFAP fluorescence density was quantified in ganglia from confocal *z*-stack images. Each image was imported into ImageJ and mean gray values of GFAP fluorescence and ganglionic area were recorded for each ganglion. GFAP fluorescence density (integrated density) was calculated as the product of area and mean gray value (McCloy et al., 2014) and is reported as the intensity in arbitrary fluorescence units (AFU) per μm^2^ of ganglionic area. Counts were performed on a minimum of 10 ganglia per animal and averaged to obtain an average packing density for that animal. *N* values represent the number of animals in each experiment.

### Statistical Analysis

Prism 6 software (Graphpad, La Jolla, CA, USA) was used for all statistical analyses and the generation of graphs. Data was analyzed using either a two-way ANOVA (with Bonferroni *post hoc* test) or a Student’s *t*-test, where appropriate. A *P* value less than 0.05 was considered statistically significant. All data are presented as mean ± standard error of the mean (SEM).

## Results

### Expression of Sirtuin-3 in the Mouse Intestine

**Figure 1:**
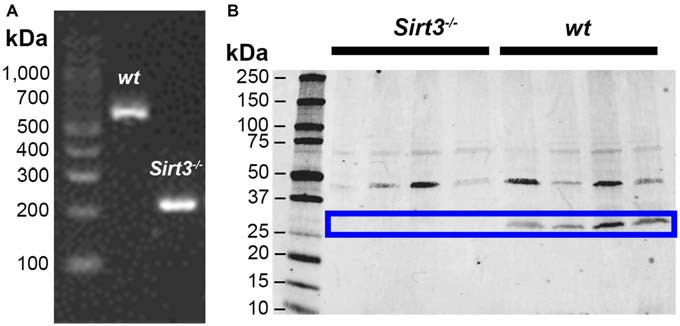
**Representative images showing detection of Sirt3 in the mouse colon by RT-PCR (A) and western blot (B).** Tissue from *Sirt3^−/−^* mice was used as a negative control. PCR amplification of Sirt3 mRNA **(A)** shows the expected products at 562 bp for *wt* *Sirt3* and 200 bp for mutant *Sirt3* (*Sirt3*^−/−^). Western blots for Sirt3 protein **(B)** show two bands potentially corresponding to the full-length nuclear protein at 44 kDa and the processed mitochondrial form at 28 kDa. However, only the band at 28 kDa is absent in *Sirt3*^−/−^ mice and this band corresponds to the single known form of mouse Sirt3.

Sirtuin-3 (Sirt3) is highly expressed in metabolically active tissues that are rich in mitochondria such as the brain and the heart (Lombard et al., 2007). Sirt3 is also expressed in the stomach (Shi et al., 2005), but whether Sirt3 expression extends to the distal regions of the gastrointestinal tract is not known. Therefore, we began by exploring the expression of Sirt3 in samples of mouse colon using PCR to assess the presence of Sirt3 mRNA and western blots to determine the presence of Sirt3 protein (Figure 1). Our results show robust expression of both Sirt3 message (Figure 1A) and protein (Figure 1B) in the colons of *wt* mice. Human Sirt3 is expressed in two forms: a long form of ~44 kDa that localizes to the nucleus and a short form of ~28 kDa that is localized to mitochondria (Schwer et al., 2002). Most available evidence shows that mice only express one form of Sirt3 that is equivalent to the human truncated form (~28 kDa; Scher et al., 2007). However, some evidence suggests that mice express both long and short forms of Sirt3 (Sundaresan et al., 2009). Our results show clear bands at both 44 and 28 kDa in samples from *wt* mouse colon (Figure 1B). However, only the band at 28 kDa was absent in samples from *Sirt3*^−/−^ mice. These results agree with the conclusion that only one form of Sirt3 is present in the mouse intestine and that this form corresponds to the truncated (28 kDa) product in humans.

### Localization of Sirtuin-3 within the Enteric Nervous System

**Figure 2:**
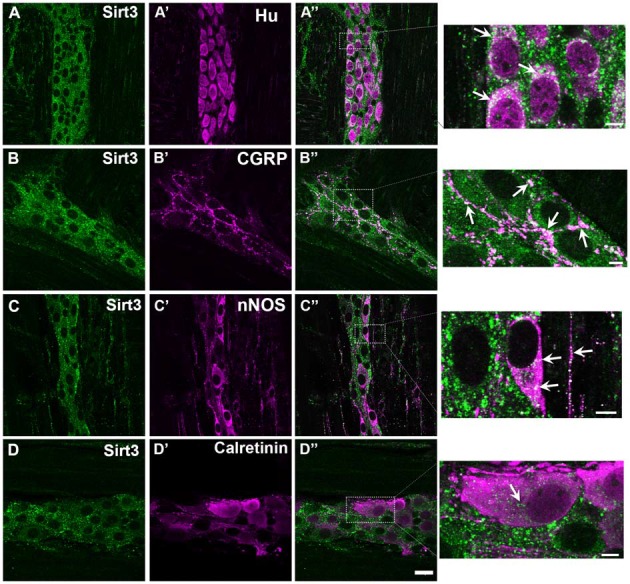
**Myenteric neurons in the mouse colon express Sirt3.** Panels show confocal fluorescence microscopy images of dual-label immunohistochemistry for Sirt3 (green, **A–D**) and either the neuronal marker HuC/D (magenta, **A’**), calcitonin gene-related peptide (CGRP; magenta, **B’**), nNOS (magenta, **C’**) or calretinin (magenta, **D’**). Overlays of each combination are shown in **(A”–D”)**. The areas demarcated by the dashed boxes in the overlay images are shown as enlarged images in the panels at far right. Arrows in these images highlight several regions of co-localization. The scale bar in **D”** = 20 μm and applies to **(A–D”)**. All scale bars in the enlarged images at right = 5 μm. Labeling is representative of experiments performed on tissue from a minimum of three mice.

Given that Sirt3 is highly expressed in the brain (Lombard et al., 2007) and our results show that Sirt3 is expressed in the colon (Figure 1), we reasoned that enteric neurons would be one site of high Sirt3 expression within the colon. To explore this possibility, we performed dual-label immunohistochemistry experiments with antibodies against HuC/D (to identify enteric neurons) and Sirt3 (to localize Sirt3 protein). In all, we evaluated the labeling of three commercial antibodies against Sirt3 in the ENS (see Table 1 for antibody details). The pattern of labeling produced by each of the three antibodies against Sirt3 that we tested was consistent with neuronal labeling in the ENS (for example, see Figure 2). However, given the questionable specificity of some antibodies (Baker, 2015), we performed careful control experiments with control peptides and tissue from Sirt3 null animals to validate the labeling of each antibody. Labeling was completely abolished in all cases by preadsorption with the corresponding peptide (data not shown); suggesting that each of the three antibodies is specific against the peptide that it was raised against. Despite all being specific for the corresponding peptides, only one antibody from Cell Signaling (Danvers, MA, USA) was specific for Sirt3 protein when we evaluated labeling in tissue from *Sirt3^−/−^* mice using immunohistochemistry and western blot analyses (western blot data shown in Figure 1B). The other two antibodies still exhibited strong labeling in knockout tissue. Based on these results, we concluded that the other two antibodies were not specific for Sirt3 protein and/or not suitable for our application and we chose to perform the remainder of our experiments with the antibody from Cell Signaling.

Our results show widespread Sirt3 immunoreactivity in myenteric neurons in the mouse colon (Figure 2). In our study, we were unable to find any HuC/D-immunoreactive neurons that did not also display some level of Sirt3 immunoreactivity (Figures 2A–A”). The most intense Sirt3 immunoreactivity that we observed was present in neuronal varicosities and co-localized well with labeling for calcitonin gene-related peptide (CGRP; Figures 2B–B”). Sirt3 immunoreactivity was present in neurons immunoreactive for nNOS and neurons immunoreactive for calretinin (Figures 2C,D). These results suggest that Sirt3 is broadly expressed by myenteric neurons and not confined to a particular neuronal subtype. However, Sirt3 may be expressed at different levels in subpopulations of enteric neurons because Sirt3 immunoreactivity appeared to label nitrergic neurons more intensely than calretinin-immunoreactive neurons (compare Figures 2C”,D”) and nitrergic nerve fibers at the level of the smooth muscle displayed strong immunoreactivity for Sirt3 (see magnification image of Figure 2C”).

### Effect of the Ablation of Sirtuin-3 on Gut Function

The results of our expression analysis above show that Sirt3 is highly expressed by myenteric neurons in the mouse colon. Based on these data, we hypothesized that a loss of Sirt3 would disrupt the normal neural control of gut function and/or the susceptibility of enteric neurons to pathological conditions that involve oxidative stress, such as inflammation. We began to test our hypothesis by assessing how the loss of Sirt3 in *Sirt3^−/−^* mice impacts the neuronal control of gut function. We assessed gut function in two ways: first, by performing isometric muscle tension recordings in isolated segments of intestine and second, by measuring colonic output *in vivo*.

**Figure 3:**
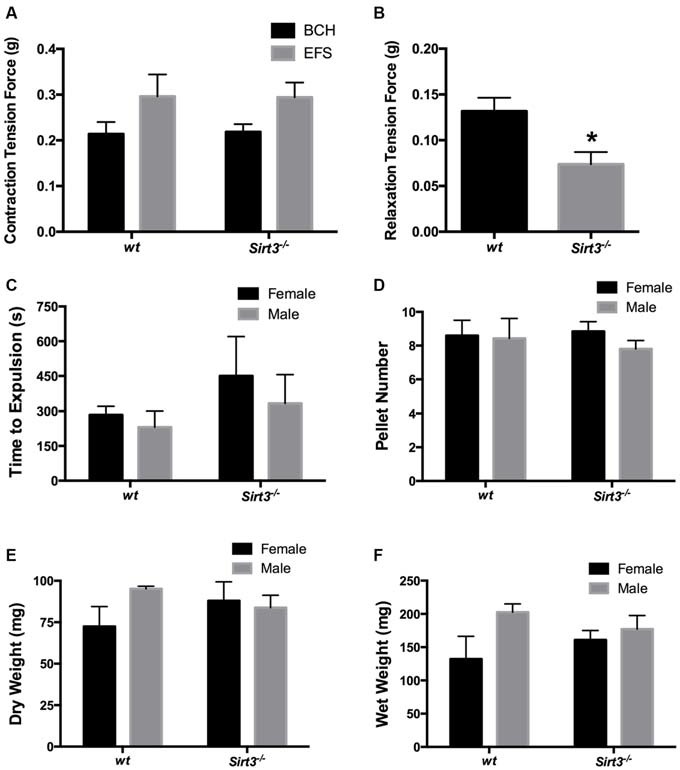
**Effect of the ablation of *Sirt3* on gut function in healthy animals. (A)** Contractile responses of longitudinal segments of colon driven by bethanechol (BCH, 10 μM) or electrical field stimulation (EFS) are similar in mice with wild type *Sirt3* (*wt*) and *Sirt3* null mice (*Sirt3*^−/−^; *n* = 5 animals per group, *P* > 0.05, Student’s *t*-test). **(B)** Relaxations driven by EFS (at 20 Hz) in pre-contracted (PGF_2α_, 5 μM) longitudinally-oriented segments of colon from *Sirt3*^−/−^ mice and their respective *wt* controls. EFS produced significantly smaller relaxations in tissues from *Sirt3*^−/−^ mice than *wt* controls (*n* = 5 animals per group, *P = 0.0192, Student’s *t*-test). Distal colonic transport time **(C)**, the number of fecal pellets produced per hour **(D)** and pellet composition **(E,F)** are comparable between male and female agouti *wt* and *Sirt3*^−/−^ mice (*n* = 4 animals per group; *P* > 0.05, 2-way ANOVA).

We began our muscle tension recordings by assessing the contractile ability of each segment of colon with the muscarinic agonist, bethanechol (BCH, 10 μM). BCH elicited similar contractile responses in tissue segments from both *wt* and *Sirt3*^−/−^ mice (Figure 3A). On average, these responses were 0.214 ± 0.026 g in *wt* and 0.218 ± 0.017 g in *Sirt3*^−/−^ tissues (*P* > 0.05). These data show that the contractile capacity of the intestinal smooth muscle is not affected by the ablation of *Sirt3*. Next, we tested whether the ability of enteric neurons to drive contractions is affected by the ablation of *Sirt3* by assessing neurogenic contractions driven by EFS. EFS elicited contractile responses of equal magnitude in tissue segments from both *wt* and *Sirt3*^−/−^ mice (Figure 3A). On average, EFS-evoked responses were 0.296 ± 0.048 g in *wt* and 0.294 ± 0.032 g in *Sirt3*^−/−^ tissues (*P* > 0.05). Overall, we found no evidence of altered neurogenic contractions between *wt* and *Sirt3*^−/−^ tissue (Figure 3A). Next, we assessed neurogenic relaxations provoked by EFS in tissue samples pre-contracted with prostaglandin F_2α_ (PGF_2α_, 5 μM; Figure 3B). These experiments revealed a significant defect in neurogenic relaxations driven by frequencies between 3–25 Hz in tissues from *Sirt3*^−/−^ mice (Data shown: EFS at 20 Hz elicited relaxation responses that averaged 0.132 ± 0.015 g in *wt* and 0.074 ± 0.013 g in *Sirt3*^−/−^ tissues; *n* = 5, *P* = 0.0192; Figure 3B). These results suggest that Sirt3 is important in the normal functions of inhibitory neurons in the ENS but is dispensable in the normal functions of excitatory neurons.

Next, we tested the hypothesis that the deficit in the inhibitory component of neuromuscular control that we observed *in vitro* would translate to poor colonic motility *in vivo*. We tested our hypothesis by assessing distal colonic transit time and by measuring the fecal pellet output rate and content in *Sirt3*^−/−^ and *wt* mice (Figures 3C,D). Our results show that colonic transit time is not significantly affected by the deletion of *Sirt3* in either male or female mice (Figure 3C). Likewise, *Sirt3*^−/−^ mice produce an equal number of pellets per hour as *wt* mice (*wt* female 9 ± 0.9 pellets, *Sirt3*^−/−^ female 9 ± 0.6 pellets, *wt* male 8 ± 1.2 pellets, *Sirt3*^−/−^ male 8 ± 0.5 pellets; *P* = 0.833; 2-way ANOVA; Figure 3D). The pellets produced contained a similar content of fecal matter and liquid (total dry weight of fecal matter: *wt* female 72.4 ± 12 mg, *Sirt3*^−/−^ female 87.9 ± 11 mg, *wt* male 95.1 ± 1.6 mg, *Sirt3*^−/−^ male 83.8 ± 7.5 mg; *P* = 0.390; 2-way ANOVA; total wet weight of fecal matter: *wt* female 132 ± 34 mg, *Sirt3*^−/−^ female 160 ± 14 mg, *wt* male 203 ± 12 mg, *Sirt3*^−/−^ male 177 ± 20 mg; *P* = 0.940; 2-way ANOVA; Figures 3E,F). These results show that the defect in neurogenic relaxations that we observed *in vitro* is not a significant barrier to normal colonic transit *in vivo*.

### Effects of the Ablation of Sirtuin-3 on Enteric Neurodegeneration During Gut Inflammation

**Figure 4:**
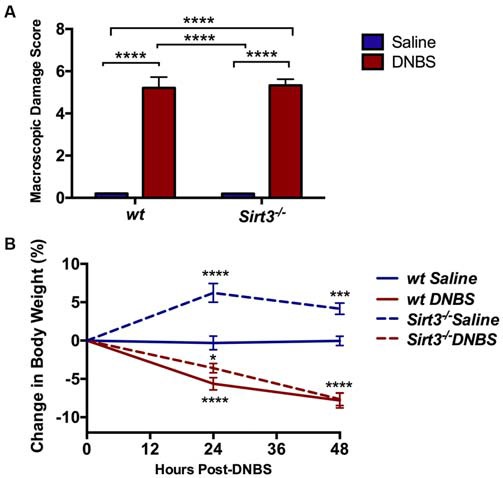
Assessment of gross inflammation driven by dinitrobenzene sulfonic acid (DNBS)-colitis in *wt* or *Sirt3^−/−^* mice as measured by macroscopic damage scoring (A) and monitoring changes in body weight (B; *n* = 5–7 per group, *P < 0.05, ***P < 0.005, ****P < 0.001, 2-way ANOVA).

Colonic inflammation increases oxidative stress in myenteric neurons and myenteric oxidative stress contributes to neurodegeneration during inflammation (Brown et al., 2016). Sirt3 plays a prominent role in the protection of central neurons from oxidative stress and decreasing Sirt3 expression increases their susceptibility to apoptosis (Kim et al., 2011; Dai et al., 2014). Therefore, we expected that mice deficient in Sirt3 would be more susceptible to myenteric neurodegeneration during inflammation. We tested our hypothesis using the DNBS model of colitis because we, and others, have shown that this model drives myenteric neurodegeneration through mechanisms that involve neuronal oxidative stress (Gulbransen et al., 2012; Roberts et al., 2013; Brown et al., 2016). DNBS drove colitis to an equal extent in both *wt* and *Sirt3*^−/−^ mice (Figure 4). Loss of *Sirt3* did not affect the extent of macroscopic tissue damage (Figure 4A) nor did it significantly change the pattern of weight loss experienced by the mice during the acute episode of colitis (Figure 4B). We did observe greater weight gain in non-inflamed *Sirt3*^−/−^ mice than healthy *wt* mice over the course of the experiment (Figure 4B) and this observation is in line with published data showing that *Sirt3*^−/−^ mice gain weight more rapidly than *wt* mice (Hirschey et al., 2011). We also observed enlarged stomachs in all mice exposed to DNBS regardless of their sex or genotype (data not shown). This observation was surprising because we have never observed this phenomenon in DNBS-colitis experiments with C57BL/6 mice (see Gulbransen et al., 2012; Brown et al., 2016). However, we did not pursue this finding further because it was independent of Sirt3 and beyond the scope of the current study.

**Figure 5:**
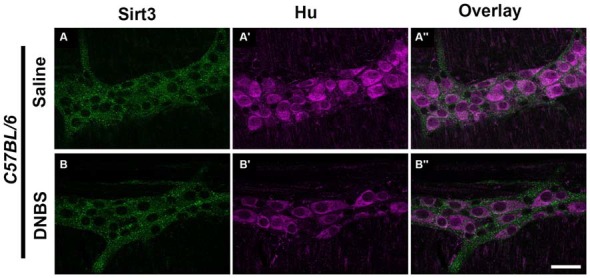
**Sirt3 expression in healthy and inflamed C57BL/6 mice**. Panels show confocal fluorescence microscopy images of dual-label immunohistochemistry for Sirt3 (green, **A,B**) and the neuronal marker HuC/D (magenta, **A’,B’**). Overlays of each combination are shown in **(A”,B”)**. The scale bar in panel **B”** = 50 μm and applies to **(A–B”)**. Labeling is representative of experiments performed on tissue from a minimum of four mice.

**Figure 6:**
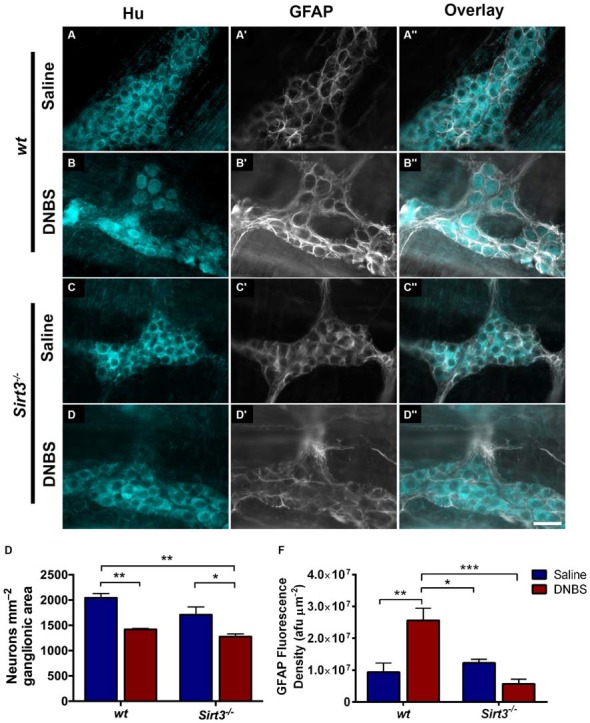
**Enteric neuron survival and glial reactivity in healthy and inflamed *wt* and *Sirt3^−/−^* mice. (A–D”)** show dual-label fluorescence immunohistochemistry for HuC/D (cyan, **A–D**) and GFAP (grayscale, **A’–D’**) in myenteric ganglia from saline- **(A–A”, C–C”)** or DNBS-treated **(B–B”, D–D”)** *wt* **(A–B”)** or *Sirt3*^−/−^ **(C–D”)** mice. Overlays are shown in **(A”–D”)**. Scale bar in **D”** = 50 μm and applies to all images. **(E)** Quantification of myenteric neuron packing density. **(F)** Quantification of ganglionic GFAP fluorescence density (afu/μm^2^). *n* = 3–5 animals per group, *P < 0.05, **P < 0.01, ***P < 0.001, 2-way ANOVA.

**Figure 7:**
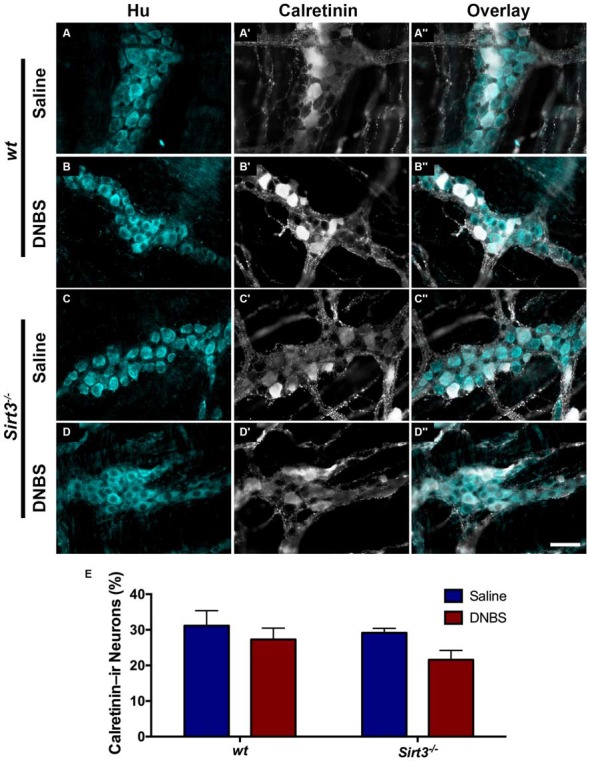
**The percentage of myenteric neurons that express calretinin is not altered by DNBS-colitis in *wt* or *Sirt3^−/−^* mice. (A–D”)** show dual-label fluorescence immunohistochemistry for HuC/D (cyan, **A–D**) and calretinin (grayscale, **A’–D’**) in myenteric ganglia from saline- **(A–A”, C–C”)** or DNBS-treated **(B–B”, D–D”)** mice. Overlays are shown in **(A”–D”)**. Scale bar in **D”** = 50 μm and applies to all images. **(E)** The percentage of HuC/D immunoreactive neurons that also display immunoreactivity for calretinin in healthy and inflamed mice with *wt* or *Sirt3*^−/−^ genotypes (*n* = 4–5 animals per group, *P* > 0.05, 2-way ANOVA).

**Figure 8:**
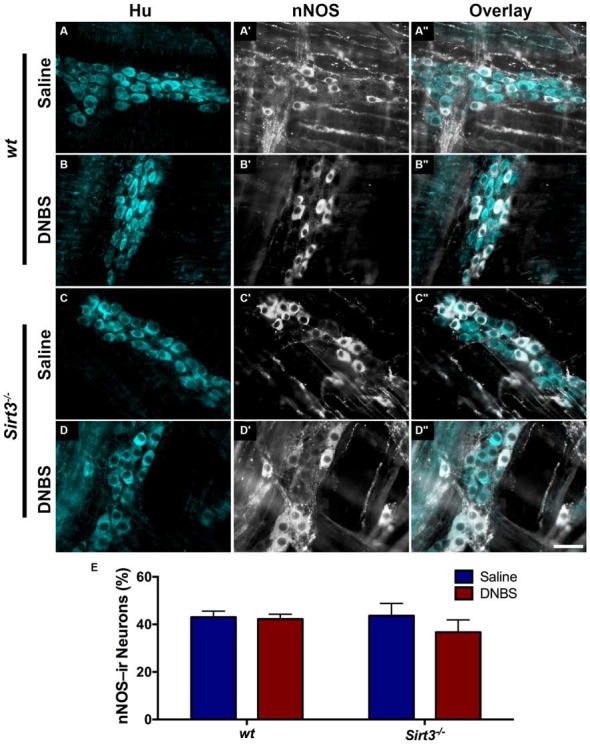
**The percentage of myenteric neurons that express nNOS is not altered by DNBS-colitis in *wt* or *Sirt3^−/−^* mice. (A–D”)** show dual-label fluorescence immunohistochemistry for HuC/D (cyan, **A–D**) and nNOS (grayscale, **A’–D’**) in myenteric ganglia from saline- **(A–A”,C–C”)** or DNBS-treated **(B–B”,D–D”)** mice. Overlays are shown in **(A”–D”)**. Scale bar in **D”** = 50 μm and applies to all images. **(E)** Quantification of the percentage of HuC/D immunoreactive neurons that also display immunoreactivity for nNOS in healthy and inflamed mice with *wt* or *Sirt3*^−/−^ genotypes (*n* = 4–5 animals per group, *P* > 0.05, 2-way ANOVA).

The results above suggest that Sirt3 is not an essential protective factor against the gross inflammatory insult driven by DNBS colitis in mice. To gain more specific insight into the role of Sirt3 in the protection of neurons, we performed whole-mount immunohistochemistry to determine if neuronal Sirt3 expression changes during inflammation and if a loss of Sirt3 alters the susceptibility to neurodegeneration and neurochemical remodeling in the myenteric plexus during inflammation. We did not detect any obvious changes in Sirt3 expression or localization within the myenteric plexus during DNBS colitis in *wt* mice (Figure 5). This outcome suggests that inflammation does not cause overt changes in Sirt3 that could account for neuronal susceptibility in the intestine. Rather, the maintenance of Sirt3 expression could be important to protect the remaining neurons. To test if the maintenance of Sirt3 expression is necessary to protect enteric neurons, we conducted inflammation studies in *Sirt3*^−/−^ mice. The packing density of HuC/D-immunoreactive myenteric neurons was not significantly different between healthy *Sirt3*^−/−^ and *wt* animals (2043 ± 85 neurons/mm^2^ in *wt* vs. 1709 ± 153 neurons/mm^2^ in *Sirt3*^−/−^, Figure 6). Likewise, the neurochemical composition of myenteric neurons was not significantly affected by the deletion of *Sirt3* because we observed equal percentages of nNOS-immunoreactive neurons (Figure 7) and calretinin-immunoreactive neurons (Figure 8) in healthy *Sirt3*^−/−^ and *wt* animals. DNBS-colitis drove myenteric neurodegeneration to a similar extent in *Sirt3*^−/−^ animals as *wt* mice (31% neuron loss in *wt* vs. 25% neuron loss in *Sirt3*^−/−^, Figure 6). This was an indiscriminate loss of myenteric neurons in both *wt* and *Sirt3*^−/−^ mice because the neurochemical makeup of the remaining HuC/D-immunoreactive neurons was not significantly different from healthy mice (Figures 7, 8). Interestingly, glia failed to up-regulate glial fibrillary acidic protein (GFAP) expression during inflammation in *Sirt3*^−/−^ mice (Figure 6F). Together, these results show that the loss of Sirt3 does not increase the susceptibility of myenteric neurons to neurodegeneration during inflammation. However, the lack of glial GFAP up-regulation suggests that *Sirt3*^−/−^ mice may experience less neuroinflammation in the myenteric plexus. Alternatively, our results indicate that Sirt3 molecular signaling might be necessary for reactive gliosis. This hypothesis should be tested in the future with cell-specific knockouts of Sirt3.

### Effects of Sirtuin-3 Inhibition on the Redox State of Enteric Neurons

**Figure 9:**
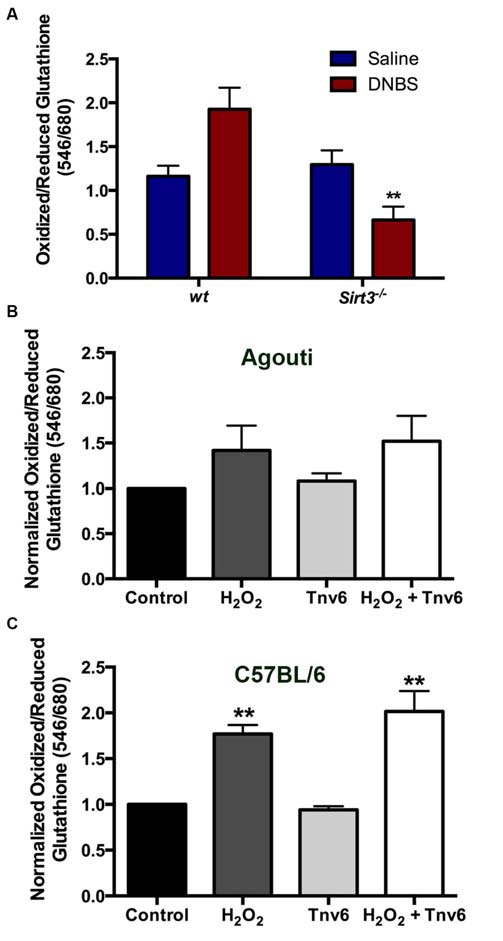
**Neither the ablation of *Sirt3* nor the inhibition of Sirt3 has a significant effect on myenteric neuron redox balance. (A)** Thiol oxidation measurements (ratio of oxidized/reduced glutathione) in myenteric neurons from saline or DNBS-treated wild type (*wt*) and *Sirt3*^−/−^ animals (*n* = 3–5 animals, **P < 0.01 vs. *wt* DNBS-treated, ANOVA). **(B,C)** Neuronal thiol oxidation measurements in whole-mount preparations from **(B)** *wt* agouti mice and **(C)** *wt* C57BL/6 mice treated with the oxidative stressor hydrogen peroxide (H_2_O_2_) and/or the Sirt3 inhibitor Tenovin-6 (Tnv-6, 100μm; *n* = 3 animals, **P < 0.01, ANOVA).

The fact that the deletion of *Sirt3* did not affect the susceptibility of enteric neurons to inflammation was very surprising because reducing Sirt3 dramatically increases neuronal vulnerability in neurons isolated from the brain (Kim et al., 2011; Dai et al., 2014; Shulyakova et al., 2014). This difference suggests that there may be important differences in the function of Sirt3 between enteric and central neurons or important differences between the roles of Sirt3 in neurons *in vivo* vs. *in vitro*. One interpretation of the above data is that Sirt3 plays a much less prominent role in the regulation of oxidative stress in enteric neurons. We tested this hypothesis by measuring how the deletion of *Sirt3* affects the redox state of enteric neurons in healthy and inflamed animals. Our results show that the ablation of *Sirt3* in *Sirt3*^−/−^ mice does not affect the oxidative balance of myenteric neurons in healthy animals (Figure 9A). In agreement with our previous work (Brown et al., 2016), myenteric neurons in tissue from *wt* mice with active DNBS-colitis exhibit higher levels of oxidative stress. However, the ablation of *Sirt3* did not exacerbate oxidative stress in enteric neurons during inflammation (Figure 9A). To the contrary, myenteric neurons from inflamed *Sirt3*^−/−^ mice exhibited less oxidative stress than neurons in tissue from healthy *wt* mice, healthy *Sirt3*^−/−^ mice or inflamed *wt* mice (Figure 9A).

The above findings were entirely unexpected and puzzling. One possible explanation for these results could be that compensatory mechanisms are enacted in the constitutive knockout animal that efficiently buffer reactive oxygen species in the absence of Sirt3. Therefore, we used a pharmacological approach to assess the effects of acutely blocking Sirt3 function with the drug Tenovin-6 (Tnv6, 100 μm). In agreement with our *in vivo* work with *Sirt3*^−/−^ mice, we found that blocking Sirt3 function acutely had no effect on the oxidative balance of myenteric neurons in tissue (Figures 9B,C). Interestingly, myenteric neurons in tissue from the *wt* agouti background mouse strain were less sensitive to oxidative stress induced by H_2_O_2_ (0.1 M) than myenteric neurons in tissue from *wt* C57BL/6 mice (Figures 9B,C). This may suggest that the handling of oxidative stress by myenteric neurons is fundamentally different in these two common mouse strains. Despite this difference, the inhibition of Sirt3 had absolutely no effect on baseline neuronal redox state or their susceptibility to an oxidative insult (driven by H_2_O_2_) in either strain. In all, these results show that Sirt3 does not play a major role in the antioxidant defenses of enteric neurons.

## Discussion

The cell autonomous mechanisms that enteric neurons utilize to regulate oxidative stress are still poorly understood. Here, we show that enteric neurons express Sirt3; a key regulator of oxidative stress in CNS neurons. Sirt3 is widely expressed by neurons throughout the myenteric plexus of the mouse colon and its expression is potentially enriched in nitrergic neurons. Our *in vivo* experiments show that Sirt3 does play a role in the normal functions of nitrergic neurons because we observed a deficit in neurogenic relaxations in tissue from *Sirt3*^−/−^ mice. However, Sirt3 seems to play a minor role, if any, in the regulation of oxidative stress by enteric neurons. Mice deficient in *Sirt3* have an incredibly unremarkable GI phenotype and do not exhibit gross abnormalities in GI function or ENS structure. Likewise, the ablation of *Sirt3* does not affect the susceptibility of enteric neurons to degeneration during colitis or their redox state.

The minor functional role of Sirt3 in the regulation of oxidative stress by enteric neurons is surprising given the major role that Sirt3 plays in the handling of oxidative stress by other populations of neurons in the CNS (Kim et al., 2011; Weir et al., 2012; Dai et al., 2014; Shulyakova et al., 2014). For example, Sirt3 expression is rapidly up-regulated by cortical neurons during excitotoxic insults and the increase in Sirt3 is an important defensive mechanism against oxidative stress that aids neuron survival (Kim et al., 2011). Sirt3 is also thought to play an important role in the regulation of oxidative stress by non-neuronal cells such as microglia (Rangarajan et al., 2015), skeletal myocytes (Jing et al., 2011), cardiomyocytes (Chen et al., 2013) and brown adipocytes (Shi et al., 2005). We did not observe any evidence of a similar role of Sirt3 in enteric neurons in our study. Enteric neurons did not exhibit higher levels of oxidative stress in *Sirt3^−/−^* mice and the acute inhibition of Sirt3 with the antagonist, tenovin-6, also had no effect on neuronal oxidative stress. These results suggest that Sirt3 fulfills a much different function in enteric neurons than in other cells and possibly that enteric neurons are fundamentally unique in the way that they handle oxidative stress. Importantly, the lack of functional Sirt3 neuroprotective mechanisms may partially explain why enteric neurons are highly susceptible to oxidative injury and death in many enteric neurodegenerative conditions (Wade and Cowen, 2004; Thrasivoulou et al., 2006; Korsak et al., 2012; Roberts et al., 2013; Brown et al., 2016).

An alternate explanation for our results is that Sirt3 is more important for the regulation of neuronal oxidative stress in culture conditions than it is *in vivo*. Indeed, the notion that Sirt3 plays a major role in the handling of neuronal oxidative stress has largely been based on data collected from cell culture model systems (Kim et al., 2011; Dai et al., 2014; Shulyakova et al., 2014). We assessed neuronal oxidative stress in tissue samples shortly after collecting them from the animal. Thus, our data exclude the possible confounding effects of the culture environment. If Sirt3 does in fact play a major role in the regulation of neuronal oxidative stress, mice lacking Sirt3 should be incredibly susceptible to neurodegeneration and would be expected to exhibit phenotypic abnormalities that reflect deficits in nervous system function. This is not the case. To the contrary, Sirt3 null mice have a surprisingly unremarkable phenotype despite having mitochondrial protein hyperacetylation at the biochemical level (Lombard et al., 2007). These discrepancies raise some interesting questions about what the primary role of Sirt3 is in the nervous system. Sirt3 undoubtedly plays an important role in the regulation of oxidative stress under some conditions but whether these conditions reflect the circumstances under which Sirt3 would normally function remains unclear.

If Sirt3 does contribute to the regulation of oxidative stress by enteric neurons, our data would suggest that this is a very minor contribution. Indeed, non-cell autonomous mechanisms mediated by enteric glia may play a more major role in the regulation of the overall redox state of enteric neurons than cell autonomous mechanism under normal conditions (Abdo et al., 2010, 2012). This major influence of glia likely masks any minor effects of the ablation of Sirt3 on the oxidative balance of enteric neurons *in vivo*. It is interesting to speculate that the same would be true of central neurons if they were assessed in their native environment where astrocytes regulate their oxidative stress vs. in culture when neuron autonomous mechanisms would be isolated and appear more prominent.

In conclusion, our study provides the first evidence of Sirt3 expression by enteric neurons. Sirt3 has received a great deal of attention as a key regulator of metabolism (Nogueiras et al., 2012) and neuroprotection against oxidative stress (Yin et al., 2015). Indeed, decreased Sirt3 expression is now being proposed by some as a potential biomarker for disease (Yu and Sun, 2015). However, we find very little evidence that would support the conclusion that low Sirt3 expression increases the susceptibility of the ENS, or the colon in general, to disease. Rather, our results are more in line with the conclusion that neuronal Sirt3 contributes very little to the overall regulation of neuronal oxidative stress and that non-cell autonomous mechanism are more important. Understanding the precise function of Sirt3 in enteric neurons will be an important issue for future molecular studies to dissect because we show that it plays a role in the normal functions of inhibitory neurons. Given the important role of Sirt3 in mitochondrial protein acetylation, a more thorough understanding of the targets and activity of Sirt3 in enteric neurons might be important to understand neuronal vulnerability or function in the gut.

## Author Contributions

BDG was responsible for the overall project conception, design and supervision. Experiments performed by RKB, IAMB, DEF and JLM. Manuscript written by RKB and BDG with all authors contributing to editing.

## Funding

This work was funded by the Crohn’s and Colitis Foundation of America (CCFA Senior Research Award to BDG), the National Institutes of Health (R01DK103723 to BDG), start-up funds from the Michigan State University Neuroscience Program to BDG and undergraduate stipends to RKB from the Michigan State University College of Natural Science and Neuroscience Program.

## Conflict of Interest Statement

The authors declare that the research was conducted in the absence of any commercial or financial relationships that could be construed as a potential conflict of interest.

## Abbreviations

Sirt: Sirtuin
GFAP: glial fibrillary acidic protein
IBD: Inflammatory Bowel Disease
DNBS: dinitrobenzene sulfonic acid
ENS: enteric nervous system
ROS: reactive oxygen species
*wt*: wildtype.
